# Neutrophil-to-Lymphocyte Ratio and MUST-Defined Nutritional Risk as Independent Correlates of Domain-Specific Quality of Life in Cancer Patients Receiving Chemotherapy

**DOI:** 10.3390/jcm15134935

**Published:** 2026-06-25

**Authors:** Arwa S. Almasaudi, Manal Naseeb, Eram Albajri, Rana H. Mosli, Nora Trabulsi, Abdurahman Almasaudi, Rouba Khalil Naaman, Layan Adawi, Raghad Almazam, Basmah Serhan, Hebah A. Kutbi

**Affiliations:** 1 Department of Clinical Nutrition, Faculty of Applied Medical Sciences, King Abdulaziz University, Jeddah 21589, Saudi Arabiaealbajri@kau.edu.sa (E.A.); hkutbi@kau.edu.sa (H.A.K.); 2 Department of Surgery, King Abdulaziz University, Jeddah 21589, Saudi Arabia; 3 Emergency Department, King Fahd Hospital, Jeddah 23325, Saudi Arabia

**Keywords:** neutrophil-to-lymphocyte ratio, Malnutrition Universal Screening Tool, quality of life, chemotherapy, systemic inflammation, nutritional risk, cancer patients

## Abstract

**Background:** Nutritional deterioration and systemic inflammation are prevalent in cancer patients undergoing chemotherapy and may independently impair health-related quality of life (QoL). Yet their simultaneous, domain-specific contributions to QoL remain poorly characterized, particularly in Middle Eastern populations. **Methods:** This cross-sectional study included adult cancer patients receiving chemotherapy. Medical records were used to collect clinical and laboratory data. Structured interviews were conducted to assess nutritional status using the Malnutrition Universal Screening Tool (MUST) and quality of life using the EORTC QLQ-C30 questionnaire. The NLR was calculated as an indicator of systemic inflammation. Multiple linear regression models, adjusted for age, sex, cancer site, stage, and treatment cycle, were used to examine independent associations with QoL domains. **Results:** Nearly 60% of patients were at a medium-to-high malnutrition risk and 27.1% exhibited high systemic inflammation (NLR > 3). The NLR was significantly associated with greater dyspnea (B = 28.4, *p* = 0.001), and the MUST was significantly associated with greater appetite loss (B = 17.0, *p* = 0.001). Additional significant associations included the NLR with poorer physical functioning (*p* = 0.009) and role functioning (*p* = 0.012), and the MUST with nausea and vomiting (*p* = 0.039). In the multivariate analysis, the NLR showed a statistically significant overall effect on the QoL profile (*p* = 0.007), while the MUST did not (*p* = 0.281), consistent with its more domain-specific pattern. **Conclusions:** This cross-sectional study suggests that systemic inflammation and nutritional risk are associated with domain-specific quality of life among cancer patients receiving chemotherapy. The NLR and MUST may represent accessible, complementary indicators of patient vulnerability and supportive care needs. Prospective multi-center studies are warranted to validate these associations and determine their clinical utility in supportive oncology practice.

## 1. Introduction

Systemic inflammation is a defining characteristic of cancer and plays a pivotal role in driving disease progression, treatment complications, and reduced survival [1]. Cost-effective biomarkers indicating inflammatory status have gained clinical interest. The Neutrophil-to-Lymphocyte Ratio (NLR) is among the most extensively researched markers of systemic inflammation and immune imbalance [2]. Derived from routine complete blood counts, the NLR reflects the increase in neutrophils, which are associated with protumor inflammation, and the decrease in lymphocytes, related to a weakened antitumor immune response [3]. An elevated NLR is associated with worse prognosis, lower treatment effectiveness, higher toxicity, and decreased survival in various cancer types [4]. Systemic inflammation plays a central role in the connection between nutritional decline, cancer-related muscle loss, and decreased survival rates in colorectal cancer [2]. Growing evidence from systematic reviews and meta-analyses confirms the prognostic significance of the NLR across multiple cancer types [4], but research on the links between systemic inflammation, nutritional status, and health-related quality of life (QoL) in patients undergoing chemotherapy is limited.

Chemotherapy frequently leads to nutritional decline due to side effects such as anorexia, nausea, vomiting, fatigue, taste alterations, and mucositis, which collectively reduce food intake and disrupt metabolic balance [5,6,7]. Malnutrition affects approximately 40–80% of cancer patients, with the prevalence varying according to tumor type, location, disease stage, treatment modality, and the nutritional assessment method employed [5,6,8,9]. Malnutrition is linked to impaired immunity, decreased functional ability, lower treatment tolerance, increased complication risks, and longer hospital stays [5].

Although systemic inflammation and nutritional risk are frequently evaluated independently, they represent deeply interconnected physiological pathways that collectively drive cancer-related decline [2,5,10]. Within this conceptual framework, the chronic systemic inflammatory response triggered by tumor biology and cytotoxic chemotherapy acts as a critical upstream driver [1]; it alters central metabolic pathways, accelerates muscle wasting, and suppresses appetite, ultimately culminating downstream in the structural weight loss and reduced dietary intake captured by the Malnutrition Universal Screening Tool (MUST) [5,6,7,11]. Consequently, while the Neutrophil-to-Lymphocyte Ratio (NLR) serves as a dynamic, real-time cellular indicator of active immunological distress and treatment-induced stress, the MUST functions as a lagging indicator of established metabolic and macronutrient depletion [10]. Investigating both pathways simultaneously provides a comprehensive, multidimensional framework to elucidate how active physiological stress and cumulative nutritional decline concurrently and independently impair health-related quality of life (QoL) in patients undergoing chemotherapy.

Quality of life (QoL) is a vital outcome in cancer care as it reflects the broad effects of disease and treatment on physical, emotional, functional, cognitive, and social well-being [12]. Chemotherapy is associated with substantial declines in QoL, driven by treatment-related symptoms, psychological distress, fatigue, and reduced functional ability [6,13]. While a growing body of evidence confirms the individual associations between nutritional status and QoL [6,14,15,16], and between systemic inflammation and clinical outcomes including muscle loss, functional decline, and lower survival rates [2,10], the simultaneous and integrated examination of both the NLR-based systemic inflammation and MUST-based nutritional risk on patient well-being during active chemotherapy remains poorly characterized. Indeed, QoL can differ depending on cancer type, treatment approach, and healthcare setting, and remains underexplored in several regions, including the Middle East [17,18,19].

Furthermore, to date, no research has assessed nutritional risk using the MUST together with the NLR and QoL in cancer patients in Saudi Arabia. Critically, the existing evidence is overwhelmingly derived from Western populations, where dietary patterns, cancer epidemiology, and healthcare access differ substantially from those in the Middle East. Given Saudi Arabia’s distinct oncological landscape and culturally specific nutritional behaviors, existing findings cannot be readily extrapolated to this population, representing a critical gap in regional supportive care evidence. Therefore, this study aims to examine the relationships between systemic inflammation, nutritional status, and quality of life among cancer patients undergoing chemotherapy at King Abdulaziz University Hospital. The study seeks to quantify the prevalence of these conditions, explore their relationships, and assess their independent associations with quality-of-life outcomes. Understanding these associations is crucial for optimizing patient care and outcomes.

## 2. Materials and Methods

### 2.1. Study Design

This hospital-based cross-sectional observational study was conducted over a three-month period from June to August 2025 at King Abdulaziz University Hospital (KAUH) in Jeddah, Saudi Arabia. The study population comprised adult patients who were admitted to the hospital’s medical wards for cancer treatment with chemotherapy. Ethical approval was obtained from the Unit of Biomedical Ethics, Faculty of Medicine, King Abdulaziz University, Jeddah, Saudi Arabia (Registration No. HA-02-J-008, Reference N0.630-22, date 29 May 2025).

### 2.2. Study Participants and Recruitment

Eligible participants included adult patients aged 18 years or older who had a confirmed cancer diagnosis and were receiving chemotherapy at KAUH. Patients were excluded if, at the time of screening, they had documented active acute infection (e.g., febrile neutropenia, bacteremia, or pneumonia), hemodynamic instability, severe cognitive impairment precluding informed consent or questionnaire completion, declined to participate, or had incomplete or missing essential data.

Recruitment was conducted using a convenience sampling approach. Patients meeting the eligibility criteria were approached in the medical wards, and the study’s purpose was fully explained before enrollment. Written informed consent was obtained from every participant prior to data collection. All 107 patients were included in descriptive and bivariate analyses. Missing data were present for the cancer stage (15 patients, 14.0%) and chemotherapy cycle number (24 patients, 22.4%); 4 patients had both variables missing. The multiple linear regression models were therefore restricted to 72 patients with complete covariate data through listwise deletion.

### 2.3. Data Collection and Instruments

Demographic and clinical data, including age, sex, primary cancer site, stage, diagnosis, and treatment regimen, were extracted from patients’ medical records. Participant identifiers were replaced with coded numbers to ensure data confidentiality. The neutrophil and lymphocyte cell counts were extracted from hospital laboratory results.

The Neutrophil-to-Lymphocyte Ratio (NLR) was calculated as an indicator of systemic inflammation. The NLR was calculated by dividing the absolute neutrophil count by the absolute lymphocyte count obtained from pretreatment complete blood counts. An NLR cutoff value of 3 was applied to categorize patients into low and high NLR groups. This threshold is consistent with findings from previous studies, where an NLR of 3 demonstrated optimal discriminatory ability for predicting both overall survival and disease-free survival [20].

Nutritional status was assessed using the Malnutrition Universal Screening Tool (MUST) [11], which identifies adults who are underweight, at risk of malnutrition, or obese. The method involves calculating the body mass index (BMI), documenting unintentional weight loss, and evaluating the impact of acute illness on nutritional intake. Scores from these components were summed to determine an overall risk category: a score of 0 represented a low risk, 1 indicated a medium risk, and a score of 2 or more denoted a high risk of malnutrition.

Health-related quality of life (QoL) was measured using the European Organization for Research and Treatment of Cancer Quality of Life Questionnaire (EORTC QLQ-C30, Version 3.0) [21]. This validated, cancer-specific tool consists of 30 questions that assess global health status, functional domains (physical, role, cognitive, emotional, and social), and several symptom scales and single items. Each item was scored and linearly transformed to a 0–100 scale according to the EORTC scoring manual. Higher scores on functional and global health scales indicate better functioning and quality of life, whereas higher symptom scores represent more severe symptoms.

The MUST and EORTC QLQ-C30 assessments were conducted via in-person interview by trained investigators within 72 h of the same cycle. This approach ensured full understanding of questionnaire items among participants and reduced missing data. Assessments occurred at different treatment cycles across patients (range: 1–16 cycles), and the cycle number was included as a covariate to account for temporal variation. Complete blood counts for NLR calculation were obtained within 48 h before each chemotherapy cycle.

### 2.4. Sample Size Calculation

Sample size was determined a priori for multivariable linear regression using G*Power (version 3.1) [22]. Assuming a moderate effect size (f^2^ = 0.15) [23], a significance level of α = 0.05, 80% statistical power, and seven predictors per model (systemic inflammation, malnutrition status, age, sex, cancer site, cancer stage, and chemotherapy cycle number), a minimum sample of 103 participants was required. The final enrolled sample was of 107 participants; this, therefore, exceeded this threshold and provided adequate statistical power for the planned analyses.

### 2.5. Statistical Analysis

Analyses were conducted using SPSS version 24.0 (IBM, Armonk, NY, USA). Categorical data were described using frequency (%). The association between systemic inflammation and malnutrition status was evaluated using the Chi-square test. Normality of distribution for the continuous QoL variables was assessed using the Shapiro–Wilk test, which indicated skewed distributions (*p* < 0.05). Therefore, QoL variables were described using the median and interquartile range (IQR). Non-parametric tests, including the Mann–Whitney U test and the Kruskal–Wallis test, were used to examine the bivariate associations of QoL variables with systemic inflammation and malnutrition status. Significant overall Kruskal–Wallis differences were further evaluated using Bonferroni post-hoc pairwise comparisons to identify specific group differences. To examine the independent associations of systemic inflammation and malnutrition status (primary predictors) with QoL (study outcome), both predictors were entered simultaneously into each multiple linear regression model, with adjustments for age, sex, cancer site, cancer stage, and the number of chemotherapy cycles (theoretical confounders).

Regression assumptions were verified by examining the residual normality (Shapiro–Wilk), multicollinearity (variance inflation factor, VIF), and heteroscedasticity (Breusch–Pagan test); influential observations were assessed using Cook’s distance (Supplementary Table S3). To verify the robustness of our findings to this analytical choice, we conducted sensitivity analyses using the continuous NLR (Supplementary Table S2), a categorical (three-phase) coding of the cycle (Supplementary Table S4), and stratification by cancer type (Supplementary Table S1). Interaction effects (NLR × cancer stage; MUST × cycle) were tested as exploratory analyses (Supplementary Table S5). To address the multiple-testing burden across the 15 QoL outcomes, a Bonferroni-corrected significance threshold of α = 0.05/15 = 0.0033 was applied. A multivariate general linear model (MANOVA) was additionally fit with all 15 QoL outcomes as joint dependent variables (Supplementary Table S6).

## 3. Results

### 3.1. Characteristics of the Sample

The study included data from 107 cancer patients on chemotherapy. The sociodemographic characteristics of the study sample are summarized in Table 1. The majority of the patients were female (77.6%). More than half of the patients had completed high school or less (56.0%) and were with overweight or obesity (58.0%). The number of chemotherapy cycles received ranged from 1 to 16.

Approximately two-thirds of the patients were at a medium- to high-risk of malnutrition (59.8%), whereas one-third demonstrated high systemic inflammation (27.1%). The Chi-square test indicated no significant association between the systemic inflammation (defined by an elevated NLR, >3) and malnutrition risk (defined by a MUST score ≥ 2), *p* = 0.837.

### 3.2. Differences in QoL Scores According to Systemic Inflammation and Malnutrition Status

Non-parametric tests were used to evaluate differences in QoL scores across levels of systemic inflammation and malnutrition status (Table 2). Patients with high systemic inflammation had significantly higher level of dyspnea (33.3 [0.00–66.7]) compared to those with low systemic inflammation (0.00 [0.00–33.3]), *p* = 0.003. Furthermore, significant differences in appetite loss were observed across the malnutrition groups (*p* = 0.002). Post-hoc comparisons showed that patients in the high malnutrition risk group reported significantly greater appetite loss than those in the low malnutrition risk group (66.7 [0.00–100] and 0.00 [0.00–33.3], respectively), *p* = 0.001.

### 3.3. Multiple Linear Regression Analyses of Systemic Inflammation and Malnutrition Status with Quality of Life (QoL)

Multiple linear regression models, adjusted for age, sex, cancer site, cancer stage, and chemotherapy cycles, were examined to evaluate the independent association of systemic inflammation and malnutrition status with QoL (Table 3). Higher systemic inflammation was associated with poorer physical functioning (B = −16.4 [95% CI −28.6 to −4.15]) and role functioning (B = −23.6 [95% CI −41.8 to −5.38]), and greater dyspnea severity (B = 28.4 [95% CI 13.8 to 43.0]).

Malnutrition status was significantly positively associated with levels of nausea and vomiting (B = 8.23 [95% CI 0.42 to 16.0]) and appetite loss (B = 17.0 [95% CI 7.03 to 26.9]). No other significant associations were observed between the systemic inflammation or malnutrition status and the remaining QoL domains.

Subgroup Analysis by Cancer Type: Stratified analyses by cancer type showed that the MUST–appetite loss association was directionally consistent in both the breast (B = 12.89, *p* = 0.064) and non-breast (B = 20.20, *p* = 0.065) subgroups, with both estimates approaching conventional significance (Supplementary Table S1). The NLR–dyspnea association was strongest in the breast subgroup (B = 43.46, *p* < 0.001) and directionally consistent in the non-breast subgroup (B = 16.54, *p* = 0.218).

Diagnostic assessment confirmed acceptable model performance, with VIF values for the main predictors of 1.14 (NLR) and 1.08 (MUST), well below the conventional threshold of 5, heteroscedasticity in 2 of 15 models (role functioning and dyspnea) for which HC3 robust standard errors preserved the conclusions, and no Cook’s distance value exceeding the strict threshold of 1.0 (Supplementary Table S3). Under conservative Bonferroni correction (α = 0.0033), the NLR–dyspnea (*p* = 0.001) and MUST–appetite loss (*p* = 0.001) associations remained statistically significant; the NLR–physical functioning, NLR–role functioning, and MUST–nausea/vomiting associations were nominally significant (*p* < 0.05) but did not survive Bonferroni correction and are reported as exploratory. Interaction tests confirmed that the primary NLR and MUST associations were stable across cancer stages and chemotherapy phases (Supplementary Table S6). In the multivariate analysis (MANOVA; Supplementary Table S6), the NLR showed a statistically significant overall effect (Wilks’ λ = 0.561, F(15, 49) = 2.56, *p* = 0.007), while the MUST did not (Wilks’ λ = 0.726, F(15, 49) = 1.23, *p* = 0.281).

## 4. Discussion

The present study provides, to our knowledge, the first integrated evidence from Saudi Arabia demonstrating that systemic inflammation and nutritional risk are independently associated with clinically meaningful domains of health-related quality of life in cancer patients undergoing chemotherapy. Results from our study revealed that approximately half (48%) of the patients participating in our study were at high risk of malnutrition. Nutritional status was significantly related to specific QoL domains, as patients in the high malnutrition risk group experienced significantly greater appetite loss compared to those in the low malnutrition risk group. Our research also indicates that malnutrition was significantly positively associated with levels of nausea and vomiting and appetite loss after controlling for potential confounders. The study’s findings support prior evidence suggesting a substantial burden of malnutrition among chemotherapy patients, although direct comparison between studies is complicated by differences in nutritional assessment tools, cancer types, and treatment settings [24,25,26]. Our results are consistent with findings from previous studies assessing nutritional risk among cancer patients. A study in Brazil using the Patient-Generated Subjective Global Assessment tool reported a prevalence of malnutrition among cancer patients of 45.3% overall, and 55% among patients who were ≥65 years of age [24]. However, another study using the Malnutrition Screening Tool reported a lower prevalence of malnutrition risk of 36.4% [27]. Additionally, although we did not specifically examine severe malnutrition in our study, it has been previously reported that 19.3% of cancer patients experience severe malnutrition [24]. Future work may therefore consider assessing the association between severe malnutrition and QoL and identifying intervention focal points for this vulnerable group. Furthermore, studies shows that patients being treated for breast cancer reported poorer emotional functioning, which was improved in well-nourished patients, while patients with oral or oropharyngeal cancer reported poorer physical functioning [25,26]. Future studies may therefore identify QoL domains to prioritize in intervention strategies according to cancer type and assess the effect of nutritional interventions on improving these QoL domains.

Although nearly half of the study population were at risk of malnutrition, more than half were classified as overweight or obese. This finding highlights a substantial gap in nutritional education and awareness among patients, where excess body weight may be mistakenly perceived as an indicator of adequate nutritional status. Such misinterpretations can delay timely nutritional assessment and intervention. In line with the ESPEN guidelines on nutrition in cancer patients [28], these findings demonstrate that reliance on body mass index (BMI) alone is inadequate for nutritional risk assessment in oncology populations. The results strongly support the routine implementation of validated malnutrition screening tools, such as the MUST, NRS-2002, or PG-SGA, as part of comprehensive cancer care to enable early detection and targeted nutritional management.

The stratified analyses by cancer type provided additional insight into the heterogeneity of these associations. The MUST–appetite loss association was directionally consistent in both the breast and non-breast subgroups, with both estimates approaching but not reaching conventional significance, likely reflecting reduced power within smaller subsamples. The NLR–dyspnea association was strongest in the breast cancer subgroup and directionally consistent in the non-breast subgroup. These findings underscore the importance of site-specific replication in larger cohorts.

Furthermore, we found that more than a quarter of patients with cancer enrolled in our study (27.1%) had high systemic inflammation as measured by the NLR. Findings from the present study indicated a significant association between systemic inflammation and various domains of QoL. In particular, patients with high systemic inflammation had significantly higher levels of dyspnea after controlling for potential confounders. Higher systemic inflammation was also negatively associated with physical functioning and role functioning. This is in line with previous studies that have demonstrated that high NLR is associated with poor outcomes among cancer patients, although a previous study using a different measure for systemic inflammation reflecting C-reactive protein and albumin levels (the Modified Glasgow Prognostic Score) found a significant association with all QoL scales and not only dyspnea, physical functioning, and role functioning [29,30]. A comparison of the association between systemic inflammation and QoL by assessment tools is warranted. Since previous studies suggested that the strength of associations between systemic inflammation and patient outcomes vary by demographic characteristics, disease history, and cancer type, future work examining the relationship between systemic inflammation and QoL may compare the strength of associations across different patient groups [29,31].

The observed associations between systemic inflammation, nutritional status, and specific QoL domains can be explained by several biological mechanisms. Elevated NLR-driven systemic inflammation promotes skeletal muscle catabolism through pro-inflammatory cytokines (IL-6 and TNF-α), which directly impair physical functioning and increase perceived fatigue [32]. In addition, the association between high NLR and dyspnea in our study may be explained by inflammatory-mediated mechanisms, including increased vascular permeability causing pulmonary edema, cytokine-induced respiratory muscle weakness, and anemia resulting from inflammation-driven erythropoiesis suppression [33]. Similarly, malnutrition negatively impacts symptom severity, decreasing treatment tolerance and decreasing cellular immunity [28], explaining our findings concerning nausea and appetite loss. Furthermore, the independent effects of the NLR and MUST on different QoL domains observed in our regression models suggest these represent parallel pathways of cancer-related deterioration, each requiring targeted intervention to optimize patient outcomes. Interestingly, no cross-sectional bivariate association was observed between the MUST-defined nutritional risk and an elevated NLR. This independence contrasts with highly catabolic gastrointestinal or acute surgical cohorts (Almasaudi et al., 2019) and highlights a distinct temporal discordance [10]: the NLR functions as a dynamic, real-time cellular indicator of immediate chemotherapy-induced stress, whereas the MUST serves as a structural lagging indicator of cumulative metabolic depletion over time. This clinical separation was likely further widened because our cohort was heavily composed of breast cancer patients (57%), where inflammation–malnutrition coupling is typically weaker. The modest explained variance of the regression models (R^2^ = 0.07–0.27) indicates that the NLR and MUST, while statistically significant correlates, account for a meaningful but limited portion of QoL variability. This is consistent with QoL being a multifactorial construct shaped by symptom burden, psychological distress, treatment-related toxicity, social and economic factors, and unmeasured biological variables.

Several clinical implications follow from these findings. Both the NLR and MUST are low-cost, minimally burdensome tools already embedded in routine oncological practice, yet their value in clinical decision-making remains underutilized. The independent associations of the NLR and MUST with non-overlapping QoL domains support distinct, complementary clinical pathways. A high MUST score is a directly actionable indicator for referral to a clinical oncology dietitian to initiate medical nutrition therapy, including oral nutritional supplements, dietary counselling, and monitoring of weight and functional status between chemotherapy cycles. An elevated NLR, while lacking a single standardized therapeutic target, serves as a low-cost “red flag” identifying patients at higher risk of symptom burden and functional decline, prompting early supportive interventions such as proactive fatigue and pain management, physical prehabilitation, and consideration of anti-inflammatory dietary measures including omega-3 fatty acid supplementation. Used together, the MUST and NLR allow clinicians to rapidly risk-stratify patients at the start of chemotherapy and route those with both a high nutritional risk and elevated inflammation to coordinated, multidisciplinary supportive care, shifting the model from reactive symptom management to proactive, personalized care. The substantial prevalence of malnutrition risk observed despite more than half of patients being classified as overweight or obese further underscores the inadequacy of BMI-based assessment alone and reinforces the case for validated multidimensional screening tools such as the MUST as a routine component of comprehensive cancer care.

Nevertheless, several limitations to this study need to be acknowledged. The cross-sectional design precludes causal inference, allowing only the assessment of associations between malnutrition, inflammation, and quality of life. Moreover, the inclusion of different cancer types and stages may introduce unmeasured confounding and mask cancer-specific associations between inflammation, nutrition, and QoL. Although stratified analyses by cancer type confirmed that the main findings were not artifacts of the breast cancer dominance in our cohort, the smaller non-breast subgroup had limited statistical power to detect moderate effects. Future studies focused on single cancer-site cohorts are needed to confirm and refine the strength of these associations. Further, the use of convenience sampling at a single center introduces a risk of selection bias and limits the generalizability of the findings to other Saudi Arabian and Middle Eastern oncology settings. Although patients with documented active acute infection were excluded, the absence of routine C-reactive protein measurement and the possibility of undocumented chronic inflammatory conditions in the medical records mean that residual inflammatory confounding cannot be entirely ruled out. In addition, the performance status (e.g., ECOG) and a formal comorbidity index were not uniformly available in the medical records used for this convenience sample and could not be included as covariates; this represents an important limitation, and we recommend that future prospective studies capture these variables to allow more comprehensive adjustment. Furthermore, assessment timing varied across patients (ranging from cycle 1 to cycle 16), which can introduce temporal variability in inflammatory and nutritional markers. Although we statistically adjusted for chemotherapy cycle number, residual confounding from treatment duration, cumulative toxicity, or disease progression cannot be entirely excluded. In addition, the modest analytic sample limits the statistical power to detect moderate interaction effects, and these null findings should not be interpreted as definitive evidence with no moderation.

Finally, information on the use of complementary or alternative medicine (CAM), including traditional herbal preparations and dietary supplements, was not systematically collected. Given the documented high prevalence of CAM use among Middle Eastern oncology patients and its potential to modulate both the inflammatory and nutritional status, this represents a source of unmeasured confounding. Future studies should prospectively capture CAM use.

## 5. Conclusions

This study shows that NLR-based systemic inflammation and MUST-defined nutritional risk are independently associated with distinct, clinically meaningful domains of health-related quality of life in cancer patients undergoing chemotherapy. Both the NLR and MUST are low-cost markers already embedded in routine clinical practice, and their combined use could inform risk-stratified supportive care during chemotherapy. Future multi-center large-sample studies are required to confirm the present findings.

## Figures and Tables

**Table 1:** Characteristics of patients on chemotherapy (*n* = 107).

Characteristics		*n* (%)
Sex	Male	24 (22.4)
	Female	83 (77.6)
Age in years	18–34	9 (8.40)
	35–49	29 (27.1)
	50–64	51 (47.7)
	65–79	16 (15.0)
	≥80	2 (1.90)
Education status	Middle school or less	30 (28.0)
	High school	30 (28.0)
	College degree	43 (40.2)
	Graduate studies	4 (3.70)
Body Mass Index	Underweight (<18.5)	2 (1.90)
	Normal weight (18.5–24.9)	42 (39.6)
	Overweight (25.0–29.9)	36 (34.0)
	Obesity (≥30.0)	26 (24.5)
Malnutrition status	Low risk	43 (40.2)
	Medium risk	12 (11.2)
	High risk	52 (48.6)
Systemic inflammation status	Low	78 (72.9)
	High	29 (27.1)
Cancer site	Breast	61 (57.0)
	Head and neck	10 (9.30)
	Gastrointestinal	23 (21.5)
	Gynecological	4 (3.70)
	Genitourinary	3 (2.80)
	Central nervous system	1 (0.90)
	Hematological	3 (2.80)
	Skin	2 (1.90)
Cancer stage	1	5 (5.40)
	2	10 (10.9)
	3	47 (51.1)
	4	30 (32.6)

Results presented as *n* (%).

**Table 2:** Differences in quality-of-life scores and the association with systemic inflammation and malnutrition status.

Quality of Life Scales	Systemic Inflammation Status			Malnutrition Status			
	Low (*n* = 78)	High (*n* = 29)	*p* Value ^a^	Low Risk (*n* = 43)	Medium Risk (*n* = 12)	High Risk (*n* = 52)	*p* Value ^b^
Functioning Scales							
Physical	73.3 [53.3–87.9]	60.0 [43.3–80.0]	0.092	73.3 [53.3–91.7]	83.3 [61.7–98.3]	66.7 [46.7–85.0]	0.084
Role	100 [50.0–100]	66.7 [16.7–100]	0.047	100 [50.0–100]	91.7 [66.7–100]	83.3 [33.3–100]	0.678
Emotional	75.0 [50.0–91.7]	50.0 [20.8–100]	0.092	66.7 [41.7–83.3]	95.8 [79.2–100]	66.7 [41.7–89.6]	0.057
Cognitive	83.3 [66.7–100]	83.3 [41.7–100]	0.108	83.3 [50.0–100]	100 [83.3–100]	91.7 [66.7–100]	0.059
Social	66.7 [50.0–100]	66.7 [50.0–100]	0.352	66.7 [50.0–100]	66.7 [50.0–100]	83.3 [50.0–100]	0.527
Symptom scales/items							
Fatigue	55.6 [22.2–66.7]	66.7 [33.3–94.4]	0.055	55.6 [22.2–77.8]	38.9 [25.0–66.7]	55.6 [33.3–88.9]	0.362
Nausea & vomiting	16.7 [0.00–50.0]	16.7 [0.00–58.3]	0.556	16.7 [0.00–33.3]	8.33 [0.00–29.2]	16.7 [0.00–50.0]	0.363
Pain	33.3 [12.5–66.7]	50.0 [33.3–83.3]	0.075	33.3 [16.7–66.7]	25.0 [0.00–45.8]	50.0 [16.7–83.3]	0.163
Dyspnea	0.00 [0.00–33.3]	33.3 [0.00–66.7]	0.003 *	0.00 [0.00–33.3]	0.00 [0.00–0.00]	0.00 [0.00–58.3]	0.126
insomnia	66.7 [0.00–100]	66.7 [16.7–100]	0.281	66.7 [0.00–66.7]	33.3 [0.00–91.7]	66.7 [0.00–100]	0.531
Appetite loss	33.3 [0.00–66.7]	0.00 [0.00–100]	0.441	0.00 [0.00–33.3]	33.3 [0.00–66.6]	66.7 [0.00–100]	0.002 *
Constipation	0.00 [0.00–66.7]	33.3 [0.00–66.7]	0.326	0.00 [0.00–66.7]	0.00 [0.00–25.0]	33.3 [0.00–66.7]	0.180
Diarrhea	0.00 [0.00–33.3]	0.00 [0.00–33.3]	0.752	0.00 [0.00–0.00]	0.00 [0.00–58.3]	0.00 [0.00–66.7]	0.081
Financial difficulties	0.00 [0.00–0.00]	0.00 [0.00–33.3]	0.281	0.00 [0.00–33.3]	0.00 [0.00–50.0]	0.00 [0.00–0.00]	0.393
Global health status	66.7 [50.0–83.3]	66.7 [33.3–83.3]	0.429	66.7 [58.3–91.7]	75.0 [50.0–83.3]	62.5 [41.7–83.3]	0.140

Results presented as median [interquartile range]. ^a^ Mann–Whitney test was used. ^b^ Kruskal–Wallis test was used. * *p* < 0.01 for the association of systemic inflammation and malnutrition status with QoL.

**Table 3:** Multiple linear regression analyses of systemic inflammation and malnutrition status with quality of life (QoL) ^a^.

QoL Domain	R^2^	Systemic Inflammation			Malnutrition Status		
		B (SE)	*p* Value	95% CI	B (SE)	*p* Value	95% CI
Functioning Scales							
Physical	0.18	−16.4 (6.11)	0.009 *	−28.6, −4.15	−1.21 (2.87)	0.675	−6.94, 4.53
Role	0.22	−23.6 (9.10)	0.012 *	−41.8, −5.38	−2.62 (4.28)	0.543	−11.2, 5.94
Emotional	0.14	−12.5 (8.39)	0.142	−29.2, 4.30	−1.11 (3.95)	0.780	−8.99, 6.78
Cognitive	0.17	−3.36 (7.39)	0.625	−18.4, 11.1	0.77 (3.47)	0.826	−6.17, 7.71
Social	0.13	1.06 (8.24)	0.898	−15.4, 17.5	3.78 (3.88)	0.333	−3.96, 11.5
Symptom scales							
Fatigue	0.12	11.7 (8.49)	0.173	−5.25, 28.7	4.88 (3.99)	0.226	−3.10, 12.8
Nausea & vomiting	0.16	9.59 (8.32)	0.254	−7.04, 26.2	8.23 (3.91)	0.039 *	0.42, 16.0
Pain	0.14	10.5 (8.94)	0.247	−7.40, 28.3	3.05 (4.20)	0.470	−5.34, 11.5
Dyspnea	0.27	28.4 (7.32)	<0.001 **	13.8, 43.0	4.54 (3.44)	0.192	−2.33, 11.4
insomnia	0.17	16.5 (11.0)	0.139	−5.52, 38.6	5.00 (5.19)	0.339	−5.37, 15.4
Appetite loss	0.27	−4.04 (10.6)	0.704	−25.2, 17.1	17.0 (4.98)	0.001 **	7.03, 26.9
Constipation	0.07	5.52 (11.5)	0.632	−17.4, 28.4	6.80 (5.41)	0.213	−4.01, 17.6
Diarrhea	0.09	−8.04 (9.39)	0.395	−26.8, 10.7	3.35 (4.41)	0.451	−26.8, 10.7
Financial difficulties	0.12	−0.14 (7.31)	0.985	−14.7, 14.5	−3.06 (3.44)	0.376	−9.93, 3.80
Global health status	0.13	−4.06 (6.65)	0.534	−17.1, 8.98	−6.15 (3.07)	0.050	−12.3, −0.01

^a^ Primary predictor: systemic inflammation and malnutrition status (inserted simultaneously). Study outcome: QoL. Models adjusted for age, sex, cancer site, stage of cancer, and number of chemotherapy cycles. * *p* < 0.05 (nominally significant); ** *p* < 0.0033 (significant after Bonferroni correction). Abbreviations: SE, standard error; CI, confidence interval.

## Data Availability

The data that support the findings of this study are available on request from the corresponding author. The data are not publicly available due to privacy or ethical restrictions.

## Supplementary Materials

The following supporting information can be downloaded at: https://www.mdpi.com/article/10.3390/jcm15134935/s1, Table S1. Stratified multiple linear regression of QoL outcomes on the NLR and MUST score, by cancer type (breast vs. non-breast); Table S2. Sensitivity analysis: NLR modeled as a continuous variable; Table S3. Regression model diagnostics for the 15 QoL outcomes; Table S4. Multiple linear regression adjusted for chemotherapy cycle phase (categorical: initiation 1–3; intermediate 4–6; maintenance > 6); Table S5. Interaction tests: NLR × advanced cancer stage and MUST × chemotherapy cycle phase; Table S6. Multivariate analysis of variance (MANOVA) for the 15 QoL outcomes.
