# Supplementary material for: Neutrophil-to-Lymphocyte Ratio and MUST-Defined Nutritional Risk as Independent Correlates of Domain-Specific Quality of Life in Cancer Patients Receiving Chemotherapy
Source: J Clin Med. 2026 Jun 25;15(13):4935. doi: 10.3390/jcm15134935 (PMC13362411; doi:10.3390/jcm15134935)
Supplement: Supplementary file 1 [file jcm-15-04935-s001.zip › jcm-4311894-supplementary.pdf]

Supplementary Tables S1–S6

Manuscript: Neutrophil-to-Lymphocyte Ratio and MUST-Defined Nutritional Risk as Independent Correlates of Domain-Specific Quality of Life in Cancer Patients Receiving Chemotherapy

Journal: Journal of Clinical Medicine (JCM)

Table S1. Stratified multiple linear regression of QoL outcomes on the NLR and MUST score, by cancer type (breast vs. non-breast).

QoL Outcome	Subgroup	NLR B	SE	95% CI	p	MUST B	SE	95% CI	p
**Physical functioning**	Breast	-15.66	8.41	[-32.77, 1.44]	0.071	1.44	3.68	[-6.04, 8.92]	0.698
	Non-breast	-19.69	8.86	[-37.94, -1.45]	**0.035**	1.01	5.86	[-11.06, 13.08]	0.865
**Role functioning**	Breast	-47.75	13.43	[-75.08, -20.42]	**0.001**	-4.86	5.87	[-16.81, 7.09]	0.414
	Non-breast	-11.38	12.19	[-36.49, 13.72]	0.359	0.61	8.06	[-16.00, 17.22]	0.941
**Emotional functioning**	Breast	-40.06	12.38	[-65.24, -14.88]	**0.003**	0.26	5.41	[-10.75, 11.27]	0.962
	Non-breast	2.14	9.87	[-18.19, 22.48]	0.830	-5.23	6.53	[-18.69, 8.22]	0.431
**Cognitive functioning**	Breast	-17.87	11.74	[-41.75, 6.01]	0.137	-1.49	5.13	[-11.93, 8.96]	0.774
	Non-breast	-0.44	7.86	[-16.63, 15.76]	0.956	-2.14	5.20	[-12.85, 8.57]	0.684
**Social functioning**	Breast	-10.49	12.60	[-36.12, 15.14]	0.411	3.11	5.51	[-8.09, 14.32]	0.576
	Non-breast	9.96	11.02	[-12.73, 32.66]	0.375	6.48	7.29	[-8.54, 21.49]	0.383
**Fatigue**	Breast	25.12	12.32	[0.06, 50.18]	**0.049**	-1.18	5.39	[-12.13, 9.78]	0.829
	Non-breast	5.99	11.85	[-18.42, 30.40]	0.618	14.13	7.84	[-2.02, 30.28]	0.084
**Nausea and vomiting**	Breast	4.24	10.55	[-17.22, 25.70]	0.690	3.46	4.61	[-5.92, 12.85]	0.458
	Non-breast	17.14	14.72	[-13.17, 47.45]	0.255	11.74	9.74	[-8.31, 31.79]	0.239
**Pain**	Breast	21.49	13.59	[-6.15, 49.14]	0.123	2.16	5.94	[-9.93, 14.25]	0.718
	Non-breast	10.60	12.99	[-16.15, 37.36]	0.422	8.31	8.59	[-9.39, 26.01]	0.343
**Dyspnea**	Breast	43.46	7.97	[27.25, 59.66]	**<0.001**	6.14	3.48	[-0.95, 13.23]	0.087
	Non-breast	16.54	13.08	[-10.40, 43.49]	0.218	0.46	8.66	[-17.36, 18.29]	0.958
**Insomnia**	Breast	26.21	17.03	[-8.43, 60.85]	0.133	4.38	7.44	[-10.76, 19.53]	0.560
	Non-breast	23.37	13.81	[-5.07, 51.81]	0.103	10.22	9.14	[-8.60, 29.03]	0.274
**Appetite loss**	Breast	-6.19	15.41	[-37.54, 25.16]	0.691	12.89	6.74	[-0.82, 26.60]	0.064
	Non-breast	2.78	15.79	[-29.74, 35.30]	0.862	20.20	10.45	[-1.31, 41.72]	0.065
**Constipation**	Breast	13.01	17.14	[-21.90, 47.92]	0.453	7.54	7.45	[-7.64, 22.72]	0.319
	Non-breast	6.62	15.25	[-24.79, 38.02]	0.668	10.53	10.09	[-10.24, 31.31]	0.306
**Diarrhea**	Breast	0.18	14.48	[-29.29, 29.64]	0.990	7.63	6.33	[-5.25, 20.51]	0.237
	Non-breast	-10.41	12.93	[-37.05, 16.23]	0.429	-7.38	8.56	[-25.01, 10.24]	0.396
**Financial difficulties**	Breast	-7.50	11.24	[-30.37, 15.37]	0.509	-4.20	4.92	[-14.20, 5.80]	0.399
	Non-breast	3.10	9.06	[-15.56, 21.77]	0.735	-10.11	6.00	[-22.45, 2.24]	0.104
**Global health**	Breast	-10.20	9.45	[-29.43, 9.03]	0.288	-10.19	4.13	[-18.60, -1.78]	**0.019**
	Non-breast	-6.33	8.54	[-23.91, 11.25]	0.465	-3.40	5.65	[-15.03, 8.24]	0.553

Each regression was adjusted for age, sex, MUST score, cancer stage, and chemotherapy cycle number. The NLR was modeled as a binary variable (> 3 vs. ≤ 3). Sample sizes after listwise deletion: breast n = 40; non-breast n = 32.

Table S2. Sensitivity analysis: NLR modeled as a continuous variable.

QoL Outcome	binary B	95% CI	p	Continuous NLR B	SE	95% CI	p
**Physical functioning**	−16.40	[−28.6, −4.15]	**0.009**	-4.897	1.378	[-7.65, -2.14]	**<0.001**
**Role functioning**	−23.60	[−41.8, −5.38]	**0.012**	-6.855	2.070	[-10.99, -2.72]	**0.002**
**Emotional functioning**	−12.50	[−29.2, 4.30]	0.142	-3.654	1.948	[-7.55, 0.24]	0.065
**Cognitive functioning**	−3.36	[−16.9, 10.2]	0.625	-2.700	1.693	[-6.08, 0.68]	0.116
**Social functioning**	1.06	[−15.4, 17.6]	0.898	-1.967	1.912	[-5.79, 1.85]	0.307
**Fatigue**	11.70	[−5.25, 28.7]	0.173	3.396	1.968	[-0.54, 7.33]	0.089
**Nausea and vomiting**	9.59	[−6.95, 26.1]	0.254	3.077	1.915	[-0.75, 6.90]	0.113
**Pain**	10.50	[−7.36, 28.4]	0.247	3.869	2.061	[-0.25, 7.99]	0.065
**Dyspnea**	28.40	[13.8, 43.0]	**<0.001**	6.573	1.719	[3.14, 10.01]	**<0.001**
**Insomnia**	16.50	[−5.43, 38.5]	0.139	5.081	2.540	[0.01, 10.16]	**0.050**
**Appetite loss**	−4.04	[−25.0, 16.9]	0.704	0.437	2.487	[-4.53, 5.41]	0.861
**Constipation**	5.52	[−17.2, 28.3]	0.632	3.015	2.636	[-2.25, 8.28]	0.257
**Diarrhea**	−8.04	[−26.7, 10.6]	0.395	-3.121	2.178	[-7.47, 1.23]	0.157
**Financial difficulties**	−0.14	[−15.0, 14.7]	0.985	1.587	1.697	[-1.81, 4.98]	0.353
**Global health**	−4.06	[−17.1, 8.98]	0.534	-0.750	1.533	[-3.81, 2.31]	0.627

B = unstandardized regression coefficient; SE = standard error; CI = confidence interval; NLR = neutrophil-to-lymphocyte ratio. All models were adjusted for age, sex, cancer site, cancer stage, and MUST score.

Table S3. Regression model diagnostics for the 15 QoL outcomes.

QoL Outcome	Breusch–Pagan p	Shapiro–Wilk p	Heteroscedastic?
**Physical functioning**	0.505	0.015	No
**Role functioning**	**0.027**	0.004	Yes
**Emotional functioning**	0.325	0.003	No
**Cognitive functioning**	0.191	0.001	No
**Social functioning**	0.380	0.028	No
**Fatigue**	0.159	0.170	No
**Nausea and vomiting**	0.077	0.074	No
**Pain**	0.851	0.071	No
**Dyspnea**	**0.039**	<0.001	Yes
**Insomnia**	0.514	0.004	No
**Appetite loss**	0.266	0.548	No
**Constipation**	0.428	<0.001	No
**Diarrhea**	0.602	<0.001	No
**Financial difficulties**	0.112	<0.001	No
**Global health**	0.481	0.133	No

Multicollinearity assessed using variance inflation factors (VIFs); heteroscedasticity using the Breusch–Pagan test; residual normality using the Shapiro–Wilk test. Sample size: n = 107. VIF values for the main predictors were 1.14 (NLR) and 1.08 (MUST), well below the conventional threshold of 5 (values < 5 indicate no multicollinearity concern). Heteroscedasticity was detected for role functioning (p = 0.027) and dyspnea (p = 0.039); HC3 robust standard errors were applied for these outcomes, with substantive conclusions unchanged.

Table S4. Multiple linear regression adjusted for chemotherapy cycle phase (categorical: initiation 1–3; intermediate 4–6; maintenance > 6).

QoL Outcome	NLR B	SE	95% CI	p	MUST B	SE	95% CI	p
**Physical functioning**	-17.69	6.00	[-29.69, -5.70]	**0.004**	0.02	2.88	[-5.72, 5.77]	0.993
**Role functioning**	-23.76	9.25	[-42.25, -5.27]	**0.013**	-2.68	4.43	[-11.54, 6.18]	0.547
**Emotional functioning**	-13.60	8.52	[-30.63, 3.42]	0.115	-0.45	4.08	[-8.60, 7.71]	0.913
**Cognitive functioning**	-3.29	7.49	[-18.25, 11.67]	0.662	0.01	3.59	[-7.16, 7.18]	0.997
**Social functioning**	0.60	8.34	[-16.08, 17.27]	0.943	4.26	4.00	[-3.72, 12.25]	0.290
**Fatigue**	11.75	8.65	[-5.54, 29.04]	0.179	5.18	4.15	[-3.10, 13.47]	0.216
**Nausea and vomiting**	11.20	8.32	[-5.43, 27.83]	0.183	7.00	3.99	[-0.97, 14.97]	0.084
**Pain**	10.80	9.10	[-7.39, 28.99]	0.240	3.06	4.36	[-5.66, 11.77]	0.486
**Dyspnea**	28.54	7.41	[13.73, 43.35]	**<0.001**	4.35	3.55	[-2.74, 11.45]	0.225
**Insomnia**	18.20	11.13	[-4.03, 40.43]	0.107	3.94	5.33	[-6.71, 14.59]	0.463
**Appetite loss**	-3.71	10.59	[-24.86, 17.45]	0.727	16.25	5.07	[6.11, 26.39]	**0.002**
**Constipation**	4.85	11.59	[-18.31, 28.02]	0.677	6.62	5.56	[-4.49, 17.72]	0.238
**Diarrhea**	-5.88	9.21	[-24.29, 12.52]	0.525	1.37	4.41	[-7.45, 10.19]	0.757
**Financial difficulties**	1.03	7.31	[-13.57, 15.63]	0.888	-4.01	3.50	[-11.01, 2.98]	0.256
**Global health**	-4.62	6.60	[-17.82, 8.57]	0.486	-5.65	3.16	[-11.97, 0.67]	0.079

Adjusted for age, sex, cancer site, cancer stage, and cycle phase (n = 83). Cycle phase distribution: initiation n = 45 (54%); intermediate n = 23 (28%); maintenance n = 15 (18%).

The pattern of findings is consistent with the primary model (Table 3): the NLR remains significantly associated with physical functioning, role functioning, and dyspnea; the MUST remains significantly associated with appetite loss.

Table S5. Interaction tests: NLR × advanced cancer stage and MUST × chemotherapy cycle phase.

QoL Outcome	NLR × Stage B	NLR × Stage p	MUST × Cycle B	MUST × Cycle p
**Physical functioning**	-21.71	0.271	-0.45	0.519
**Role functioning**	-24.42	0.408	-0.54	0.600
**Emotional functioning**	-44.45	0.100	-0.78	0.409
**Cognitive functioning**	-42.51	0.072	-0.37	0.660
**Social functioning**	8.58	0.749	-1.18	0.202
**Fatigue**	54.68	**0.044**	-0.81	0.397
**Nausea and vomiting**	-14.05	0.603	-0.73	0.436
**Pain**	21.83	0.451	-0.50	0.623
**Dyspnea**	36.20	0.125	-0.17	0.833
**Insomnia**	34.70	0.326	0.81	0.514
**Appetite loss**	-24.16	0.482	1.75	0.142
**Constipation**	-25.77	0.484	0.28	0.828
**Diarrhea**	13.91	0.648	0.74	0.487
**Financial difficulties**	35.09	0.132	0.64	0.437
**Global health**	-11.85	0.577	-1.53	**0.036**

B = interaction coefficient. After Bonferroni correction for the 30 interaction tests ($\alpha = 0.05/30 = 0.0017$), no interaction term reached statistical significance in any model. Two nominal interactions were observed at $\alpha = 0.05$: NLR × advanced stage for fatigue ($B = 54.68$, $p = 0.044$) and MUST × continuous cycle for global health ($B = −1.53$, $p = 0.036$). These exploratory findings are consistent with the chance expectation under multiple testing (≈1.5 false positives expected at $\alpha = 0.05$ across 30 tests).

Table S6. Multivariate analysis of variance (MANOVA) for the 15 QoL outcomes.

Predictor	Wilks' λ	F	df (num, den)	p-value
**NLR (>3 vs. ≤3)**	0.561	2.56	15, 49	**0.007**
**MUST score**	0.726	1.23	15, 49	0.281
**Age**	0.595	2.23	15, 49	**0.018**
**Sex**	0.636	1.87	15, 49	0.050
**Cancer site**	0.687	1.49	15, 49	0.147
**Cancer stage**	0.838	0.63	15, 49	0.833
**Cycle number**	0.827	0.68	15, 49	0.787

Multivariate general linear model with all 15 EORTC QLQ-C30 QoL outcomes as joint dependent variables and the same predictors as the univariate regressions (NLR, MUST, age, sex, cancer site, cancer stage, and cycle number).

Wilks' lambda (λ) is the multivariate test statistic; lower values indicate stronger multivariate effects. The NLR showed a statistically significant overall multivariate effect (p = 0.007), supporting the NLR's role as a multivariate predictor of the QoL profile. The MUST did not reach multivariate significance (p = 0.281), consistent with the univariate evidence of a more domain-specific MUST effect (concentrated in appetite loss).
